# Combined elevated C-reactive protein and detectable plasma RNA has predictive value for hospitalization during an outpatient COVID-19 convalescent plasma clinical trial

**DOI:** 10.1016/j.jcvp.2026.100268

**Published:** 2026-09-09

**Authors:** Yuriko Fukuta, Atika Singh, Jiangda Ou, Leilani Montalvo, Clara Di Germanio, Moises A. Huaman, Aarthi G. Shenoy, Giselle S. Mosnaim, Bela Patel, Sonya L. Heath, Adam C. Levine, Barry R. Meisenberg, Shweta Anjan, Janis E. Blair, Judith S. Currier, James H. Paxton, Jonathan Gerber, Laura L. Hammitt, Catherine G. Sutcliffe, William Rausch, Patrick B. Broderick, Marie-Elena Cordisco, Benjamin Greenblatt, Valerie C. Cluzet, Daniel Cruser, Kevin Oei, Abinante Matthew, Donald N. Forthal, Martin S. Zand, Seble G. Kassaye, Edward R. Cachay, Jay S. Raval, Thomas J. Gniadek, Emily Spivak, Kelly A. Gebo, Shmuel Shoham, Arturo Casadevall, Nichol A. McBee, David M. Shade, Stephan Ehrardt, Oliver Layendecker, Sabra L. Klein, Evan M. Bloch, Aaron A. R. Tobian, Daniel Ford, Karen Lane, Daniel F. Hanley, Andrew Pekosz, Bryan Lau, Philip J. Norris, David J. Sullivan, CSSC (COVID 19 Serologic Studies Consortium) group

**Affiliations:** 1 Department of Medicine, Section of Infectious Diseases, Baylor College of Medicine, Houston, TX, USA; 2 Department of Neurology, Brain Injury Outcomes, Johns Hopkins University School of Medicine, Baltimore, MD, USA; 3 Vitalant Research Institute, San Francisco, California, USA; 4 Department of Medicine, Division of Infectious Diseases, University of Cincinnati, Cincinnati, OH, USA; 5 Department of Medicine, Division of Hematology and Oncology, MedStar Washington Hospital Center, Washington DC, USA; 6 Division of Allergy and Immunology, Department of Medicine, NorthShore University Health System, Evanston, IL, USA; 7 Department of Medicine, Division of Pulmonary and Critical Care Medicine, University of Texas Health Science Center, Houston, TX, USA; 8 Department of Medicine, Division of Infectious Diseases, University of Alabama at Birmingham, Birmingham, AL, USA; 9 Department of Emergency Medicine, Warren Alpert Medical School of Brown University, Providence, RI, USA; 10 Luminis Health, Annapolis, MD, USA; 11 Department of Medicine, Division of Infectious Diseases, University of Miami Miller School of Medicine, Miami, FL, USA; 12 Department of Medicine, Division of Infectious Diseases, Mayo Clinic Hospital, Phoenix, AZ, USA; 13 Department of Medicine, Division of Infectious Diseases, University of California, Los Angeles, CA, USA; 14 Department of Emergency Medicine, Wayne State University School of Medicine, Detroit, MI, USA; 15 Department of Medicine, Division of Hematology and Oncology, University of Massachusetts Chan Medical School, Worcester, MA, USA; 16 Department of International Health, Johns Hopkins Bloomberg School of Public Health, Baltimore, MD, USA; 17 Nuvance Health, Danbury, CT, USA; 18 Nuvance Health Danbury Hospital, Danbury, CT, USA; 19 Nuvance Health Norwalk Hospital, Norwalk, CT, USA; 20 Nuvance Health Vassar Brothers Medical Center, Poughkeepsie, NY, USA; 21 Ascada Research, Fullerton, CA, USA; 22 Department of Medicine, Division of Infectious Diseases, University of California, Irvine, CA, USA; 23 Department of Medicine, University of Rochester Medical Center, Rochester, NY, USA; 24 Department of Medicine, Division of Infectious Diseases, Georgetown University Medical Center Washington DC, USA; 25 Department of Medicine, Division of Infectious Diseases, University of California, San Diego, CA, USA; 26 Department of Pathology, University of New Mexico School of Medicine, Albuquerque, NM, USA; 27 Department of Pathology and Laboratory Medicine, Northshore University Health System, Evanston, IL; 28 Department of Medicine, Division of Infectious Diseases, University of Utah School of Medicine, Salt Lake City, UT, USA; 29 Department of Medicine, Division of Infectious Diseases, Johns Hopkins University School of Medicine, Baltimore, MD, USA; 30 W. Harry Feinstone Department of Molecular Microbiology and Immunology; Johns Hopkins Bloomberg School of Public Health, Baltimore, MD, USA; 31 Department of Epidemiology, Johns Hopkins Bloomberg School of Public Health, Baltimore, MD, USA; 32 Division of Intramural Research, National Institute of Allergy and Infectious Diseases, Baltimore, MD, USA; 33 Department of Pathology, Johns Hopkins University School of Medicine, Baltimore, MD, USA; 34 Institute for Clinical and Translational Research, Johns Hopkins University School of Medicine, Baltimore, MD, USA; 35 COVID 19 Serologic Studies Consortium group full listing in supplement material

**Keywords:** SARS-CoV-2, COVID-19 convalescent plasma, hospitalization, biomarkers, c-reactive protein, plasma RNA, viremia

## Abstract

**Background:**

Laboratory biomarkers that predict progression from mild COVID-19 to hospitalization are not well defined.

**Objective:**

To evaluate the performance of C-reactive protein (CRP) and plasma SARS-CoV-2 RNA for predicting hospitalization among mild COVID-19 outpatients enrolled in a randomized controlled trial of COVID-19 convalescent plasma (CCP).

**Methods:**

CRP and plasma SARS-CoV-2 RNA were measured in 487 outpatient trial participants screened before March 2021. Weighted analyses accounting for sampling probabilities were used to estimate positive predictive values (PPVs) and sensitivities for progression to hospitalization, with performance compared across trial arms.

**Results:**

Elevated CRP (>1 mg/dL) was present in 27% (131/487) of participants, and plasma SARS-CoV-2 RNA was detectable in 15% (74/487). In weighted analyses, PPVs for hospitalization were 15% (95% CI, 10–21) for elevated CRP alone, 25% (95% CI, 16–35) for detectable plasma RNA alone, and 37% (95% CI, 23–52) when both biomarkers were present. Among participants with elevated CRP and plasma RNA >500 copies/mL, PPV increased to 72% (95% CI, 37–100). Elevated CRP demonstrated 100% sensitivity for hospitalization in CCP-transfused participants and 54% in control plasma–transfused participants, while detectable plasma RNA demonstrated sensitivities of 60% and 50%, respectively. Among individuals meeting severe COVID-19 risk criteria based on sex, age, obesity, or comorbidities, the combination of normal CRP and undetectable plasma RNA have a high negative predictive value for hospital progression risk.

**Conclusions:**

The combination of elevated CRP and detectable plasma SARS-CoV-2 RNA improves laboratory-based risk stratification for progression to hospitalization among outpatients with mild COVID-19.

## 1. Introduction

Early in the COVID-19 pandemic, C-reactive protein (CRP) was among many biomarkers associated with hospitalized patient adverse outcomes[1–4]. A longitudinal COVID-19 outpatient study in 571 Icelandic individuals noted elevated CRP in 67% of people later hospitalized and 97% in those who later progressed to the intensive care unit (ICU) or died [5]. A Cochrane analysis including outpatients noted elevated CRP was 78% sensitive and 72% specific in predicting outpatient COVID-19 hospitalizations [6].

Most plasma SARS-CoV-2 RNA studies conducted prior to the Delta variant emergence and widespread vaccination, primarily examined association with mortality among already hospitalized patients [7, 8]. Plasma RNA decreased after antibody generation [9] to correlate with older age [10], obesity [11, 12] and severe disease comorbidities [13]. The SARS-CoV-2 plasma RNA detection in presymptomatic blood donors<10% [14–16], outpatients with mild disease-11% [12], emergency department presentation-23%[17] or 33%[18] and those hospitalized-34% [19] indicates an increased detectable plasma RNA likelihood with disease progression[7].

Few studies describe mild COVID-19 outpatient biomarkers within the first illness week[6, 17, 20–22]. We hypothesized that the combination of pretransfusion CRP and plasma SARS-CoV-2 RNA obtained in the first week of illness before disease progression would improve prediction for hospitalization outpatients with mild COVID-19.

## 2. Methods (Additional full detailed methods in supplement)

### 2.1 Participant population

This study is a follow-up secondary analysis to investigate how pretransfusion CRP levels and detectable plasma SARS-CoV-2 RNA predict progression to the hospitalization primary outcome. The original outpatient, double-blind, randomized controlled trial under IND 19725 compared CCP to control plasma transfusion from June 2020 through September 2021 in 1181 confirmed COVID-19 participants enrolled within 8 days of symptom onset [23]. This subsample analyzed 487 participants including 43 of the 54 hospitalizations transfused before March 2021 with available plasma for testing.

### 2.2 Study Approval

Johns Hopkins University (JHU) served as the single-Institutional Review Board and approved the IRB protocol IRB00247590 on April 28, 2020. The protocol was also independently reviewed and approved by the Navajo Nation Health Human Research Review Board (NNR-20.376) and the Indian Health Service National IRB (N20-N-06). Written and signed informed consent were obtained from all participants. The trial was registered on clinicaltrials.gov-NCT04373460. A secondary analysis protocol titled “Secondary Analysis of Outpatient COVID-19 Convalescent Plasma Studies” with IRB number IRB00415718 was also obtained.

### 2.3 SARS-CoV-2 Plasma RNA analysis

The Grifols Procleix SARS-CoV-2 transcription-mediated amplification (TMA) qualitative assay (50% LOD 2.46 copies/mL) was performed on the Panther System, using 500 μl plasma for extraction and analysis[18]. For qRT-PCR, viral RNA was extracted using QIAamp Viral RNA Mini Kit (QIAGEN). For each plasma sample, 140 μl was processed and eluted in 100 μl of water with 10 μL duplicate input into PCR or 14 μL plasma equivalents[18]. A nucleocapsid gene 73-base pair region was targeted based on an assay developed by the US Centers for Disease Control and Prevention[24].

### 2.4 Hospitalization outcomes

Hospitalization data from discharge summaries, local principal investigator summaries and data submitted to the trial COVID-19 related hospital adjudication physician panel were reviewed for oxygen requirements, length of stay, ICU admission and laboratory studies

### 2.5 Statistical analysis

Individuals who progressed to hospitalization were selected with a probability of 1, while those who were high risk participants and those who were otherwise healthy were selected with a probability of <1. Therefore, the risk of hospitalization cannot be estimated without bias unless the sample is reweighted back to represent the original 795 participants who were at risk given the eligibility criterion of transfusion prior to March 1, 2021. For all analyses that depend on risk (e.g., positive predictive value, negative predictive value, hospital risk, etc.), the analytical sample was weighted by the inverse of the probability of selection into this subsample for plasma RNA testing[25]. This accounts for differential sampling probabilities, so that results will reflect the full eligible cohort.

The statistical relationship between hospitalization status and CRP or RNA levels was estimated by a logistic model. Because of weighted analysis, we used the robust sandwich estimator for obtaining 95% confidence intervals (CI). Furthermore, there were situations in which there was complete separation of the logistic model due to no events in one level of predictor. Therefore, we utilized Firth’s penalized logistic regression (weighted by inverse sampling probability) with 2000 iterations of bootstrap approach [26]. Analyses were performed using R and GraphPad Prism 8 (GraphPad Software).

## 3. Results

### 3.1 Participant sample

This 487-participant plasma RNA subgroup analysis of the completed full trial 1181 participants randomly transfused CCP or control plasma was selected to include hospitalizations (n=43) and a random selection of transfusion participants (n=444) before March 1, 2021, in both those healthy (n=58) and those with severe COVID-19 risk factors (n=429) (Table 1) and Figure 1. The time period of the substudy before March 1 and participant risk factors for severe disease progression is a similar match to the emergency room enrollment study called C3PO[27] which also analyzed plasma viral RNA[18]. Full trial participants with any risk factor for severe COVID-19 was 66% (782/1181), lower than the 88% (429/487) in this subsample analysis. Those hospitalized comprised 95% (41/43) with severe COVID-19 risk factors driven principally by higher age (≥50 years) participants. This RNA subsample analysis comprised 86% seronegative participants and no fully vaccinated participants. Roughly half were transfused within 5 days of symptom onset in participants not hospitalized. Transfusions within 5 days of symptom onset occurred in 27% (4/15) hospitalized after CCP compared to 75% (21/28) after control plasma. CCP reduced participant hospital progression to 6.2% (15/242) compared to the control plasma group 11.4% (28/245) (p=.042) and had greater reduction when given early.

### 3.2 CRP and Plasma RNA detection by PCR

An elevated participant CRP ≥1 mg/dL (=10 mg/L) was present in 28% (322/1149) prior to transfusion. The same non-weighted proportion was seen in the 487 plasma RNA subgroup, where an elevated CRP was present in 27% (131/487) (Table 2). Participant plasma RNA by PCR was detected in 15% (74/487). Risk factors for plasma RNA were BMI ≥ 30, age over 50 years, diabetes, hypertension, elevated CRP, and comorbidities (Table 3). The ratio of participants with elevated CRP, positive PCR, and both results segregated by interval from symptom onset to transfusion shows elevated CRP average 25% during the first 6 days, then increasing to 35% (Figure 2A). Within the first three days 24% of subjects had detectable plasma RNA, decreasing to 16% on day 4 and 5 and to 9% by days 6-9. Those participants with both high CRP and detectable plasma RNA were constant at 8-10%. While enrollment nasal viral load copy number decreased by 2 logs (million to 10,000) with longer interval from symptoms onset, the plasma viral load remained level at 100 copies/mL (Figure 2B). There was no significant correlation between nasal viral load and plasma viral load (Spearman correlation ≥ 0.05) (supplement Fig 1).

### 3.3 Subsequent hospitalization with elevated CRP and plasma RNA detection

In the full trial where CRP was measured in 1149 participants pretransfusion in the first week of illness, 40 of 54 participants subsequently hospitalized had a screen CRP≥1 for a 74% sensitivity. The hospitalization PPV for elevated CRP was 12% (40/322) with a 98.3% (813/827) negative predictive value (NPV) (Table 2). Within the non-weighted subsample analysis, a high pretransfusion CRP was present in 30 of 43 hospitalizations (all 15 CCP and 15 of 28 control plasma hospitalizations). A positive pretransfusion plasma RNA was present in 53% (23/43) hospitalizations (9/15 CCP and 14/ 28 control plasma hospitalizations) (Table 3).

### 3.4 Weighted analysis positive predictive value for subsequent hospitalization

The overall participant population transfused before March 1, 2021, was 795. Sampling probabilities were used to reweight the 487-participant subsample back to the original 795 sample to estimate hospital risk or PPV. The 43 hospitalized were all selected (P=1). Of the 752 potential nonhospitalized participants, 56/232 among the healthy non-hospitalized participants were selected (P=0.24) and 388/520 among age≥50, obese, or other co-morbidities were also selected (P=0.75). The analytical participant sample was weighted by the inverse of the selection probability into this plasma RNA subsample.

In the weighted analysis of both intervention groups together, elevated CRP showed a 15% PPV (30/194; [95%CI:10-21]) compared to non-weighted PPV 23% (Table 2 and Fig. 3A-C). CRP sensitivity was 70% (30/43). The weighted analysis for detectable plasma RNA indicated a higher weighted 25% PPV (23/91; [95%CI:16-35]), a non-weighted 31% PPV and a CRP with lower sensitivity 53% (23/43). Combining detectable plasma RNA and elevated CRP increased PPV to 37% (18/49; [95%CI:23-52]) with sensitivity of 60% (18/30). The small group with PCR copy number ≥500 showed a hospital progression of 72% PPV (7/9.7; [95%CI:37-100]).

In the control plasma group segregating the two tests into the weighted intervention arms, PPV for elevated CRP was 16% (15/95; [95%CI:8-25]) with sensitivity 54% (15/28), while detectable plasma RNA PPV rose to 36% (14/39; [95%CI:20-53]) with sensitivity 50% (14/28). Addition of plasma RNA testing to elevated CRP increased PPV to 49% (9/18.4; [95%CI:24-76]) which rose to 100% (5/5; [95%CI:100-100]) in the 5 individuals with high plasma RNA copy number equal or greater than 500/mL.

In the CCP group the detectable plasma RNA PPVs was nearly 50% reduced. While elevated CRP had a similar CCP to control PPV at 15% (15/99; [95%CI:8-23]), the sensitivity was 100% (15/15). Detectable plasma RNA CCP PPV fell to 17% (9/51.7; [95%CI:8-29]) from control at 36%, with sensitivity 60% (9/15). Addition of plasma RNA testing to elevated CRP decreased CCP PPV to 30% (9/30; [95%CI:14-48]) compared to control arm 49%. High plasma RNA copy number 500 or over was 43% (2/4.7) CCP PPV down from 100% PPV in the control arm.

A weighted analysis for PPV by increasing CRP from <0.5, 0.5 to .99, 1-3.99 and >4 mg/dL indicated zero PPV in the CCP arm with CRP less than 1 and small difference in CRP 1-3.99, and no difference in high CRP 4 or over between arms (Fig. 3D, E). For the plasma RNA, negative detection was a 4 to 2 PPV reduction, while the increasing plasma RNA copy numbers from 1-150, 151 to 499 and 500 or higher indicated 50% reductions in the PPV with increasing copy number.

### 3.5 Non-weighted trial arm hospital outcome differences

The non-weighted comparison of COVID-19–related hospitalization participants in the CCP group (n=15) versus control plasma group (n=28) transfused by March 1, 2021, noted similar age, BMI and comorbidities, except for a higher percent and mean elevated CRP in the CCP group and lower number transfused within 5 days of symptom onset (Table 1 and 4). Subsequent biomarkers measured during hospital admission outside of the study protocol and obtained from study hospital COVID-19 adjudication materials indicated higher levels of CRP, D-dimer, and ferritin in the CCP than the control group, although only the difference in ferritin was statistically significant. While 25% (n=7) of the control group required mechanical ventilator management, bilevel positive airway pressure or high flow oxygen, only 13% (n=2) of CCP group required bilevel positive airway pressure or high flow oxygen (P=.61). More participants in the control plasma group received COVID-19 convalescent plasma, remdesivir and dexamethasone treatments during hospitalization than the CCP group, although none of these differences were statistically significant. Length of stays were the same in both groups. More participants in the control plasma group, 36% (9/28) versus CCP group-13% (2/15), required home supplemental oxygen upon discharge.

## 4. Discussion

Our results suggest that elevated CRP values combined with the presence of plasma viral RNA are associated with a higher hospitalization progression, thus identifying individuals for antiviral intervention. Plasma viral RNA predicting severe disease applies to MERS[28], SARS-CoV-1[29], Ebola[30], H5N1 influenza[31]. Although our study evaluated only SARS-CoV-2 infection, plasma RNAemia may represent a broader marker of systemic viral dissemination and host inflammatory activation[7]. A recent systematic review of respiratory viral infections found that viraemia has been reported in SARS-CoV, MERS-CoV, influenza, RSV, adenovirus, rhinovirus, and other respiratory viruses, with many studies demonstrating an association between viraemia and disease severity, although the strength of evidence varies among pathogens[19]. Accordingly, our findings should not be interpreted as demonstrating applicability to these infections, but rather as supporting the hypothesis that combined virologic and inflammatory biomarkers may warrant evaluation across emerging respiratory viral diseases.

We report that the PPV for hospitalization doubled in those with both a high CRP and detectable plasma RNA with sensitivity in the 60% range compared to elevated CRP alone. Conversely, normal CRP and undetectable plasma RNA conferred a 99% negative predictive value (NPV) for hospitalization! Viremia in respiratory viral infection varies by virus, which presumably reflects larger lung burdens[19]. Nasal SARS-CoV-2 viral loads in mild outpatient COVID-19 patients modestly predicted hospital progression in ACTIV-2[32], but not in emergency department patients[33]. Potential implications of these findings could help 1) identify individuals who would benefit the most from outpatient therapeutic interventions; 2) identify those who may need closer monitoring/surveillance for progression to severe disease. The NPV is quite high for both tests.

This subsample analysis focused on unvaccinated participants in the prealpha period, of whom 85% were seronegative at the pretransfusion visit. This plasma SARS-CoV-2 RNA analysis may have benefited from both restrictive definition of COVID-19 related hospitalizations and more restrictive plasma RNA definition.

We also chose a binary CRP biomarker analysis rather than a receiver operating curve biomarker analysis for a greater translation potential to the clinic where a widely available CRP determination[2] can be followed by a plasma PCR if CRP is high. Those who were doubly positive, were at double the risk for hospitalization within the subsequent week in a population where 71% (weighted) had any risk factor (age, obesity or comorbidities) for progression to hospitalization. Our approach ignores potential continuous relationships between biomarker levels and risk, which could provide finer risk stratification. Plasma PCR may be more widely available on a short time scale to implement this two-step hospital risk prediction for outpatients.

The non-weighted comparison for differential outcomes after the COVID-19 related primary outcome was limited due to the small numbers in the two intervention groups (CCP-15, control plasma-28). Notable observed trends in the CCP group versus control were no deaths or mechanical ventilation, shorter duration of hospitalization, smaller percent requiring home oxygenation at discharge.

In summary, this subsample analysis correlated both high CRP and detectable plasma viral RNA with a doubling of the PPV for hospitalization. The results have biological COVID-19 pathogenesis plausibility since severe COVID-19 symptoms reflect increased inflammation, which in turn is associated with higher CRP levels and can be triggered by a higher viral lung burden. The processing time for SARS-CoV-2 nasal viral load determination and plasma RNA is similar. In the current 2025 setting where 97% of the population is seropositive for SARS-CoV-2, this two-step biomarker approach to severe disease prediction is more relevant in the case of greater outpatient severe disease progression or higher case fatality rates in those seronegative for future pandemics. Among individuals meeting severe COVID-19 risk criteria based on sex, age, obesity, or comorbidities, the combination of normal CRP and undetectable plasma viral RNA have a high NPV for hospital progression risk.

## Supplementary Material

1

## Figures and Tables

**Figure 1.**
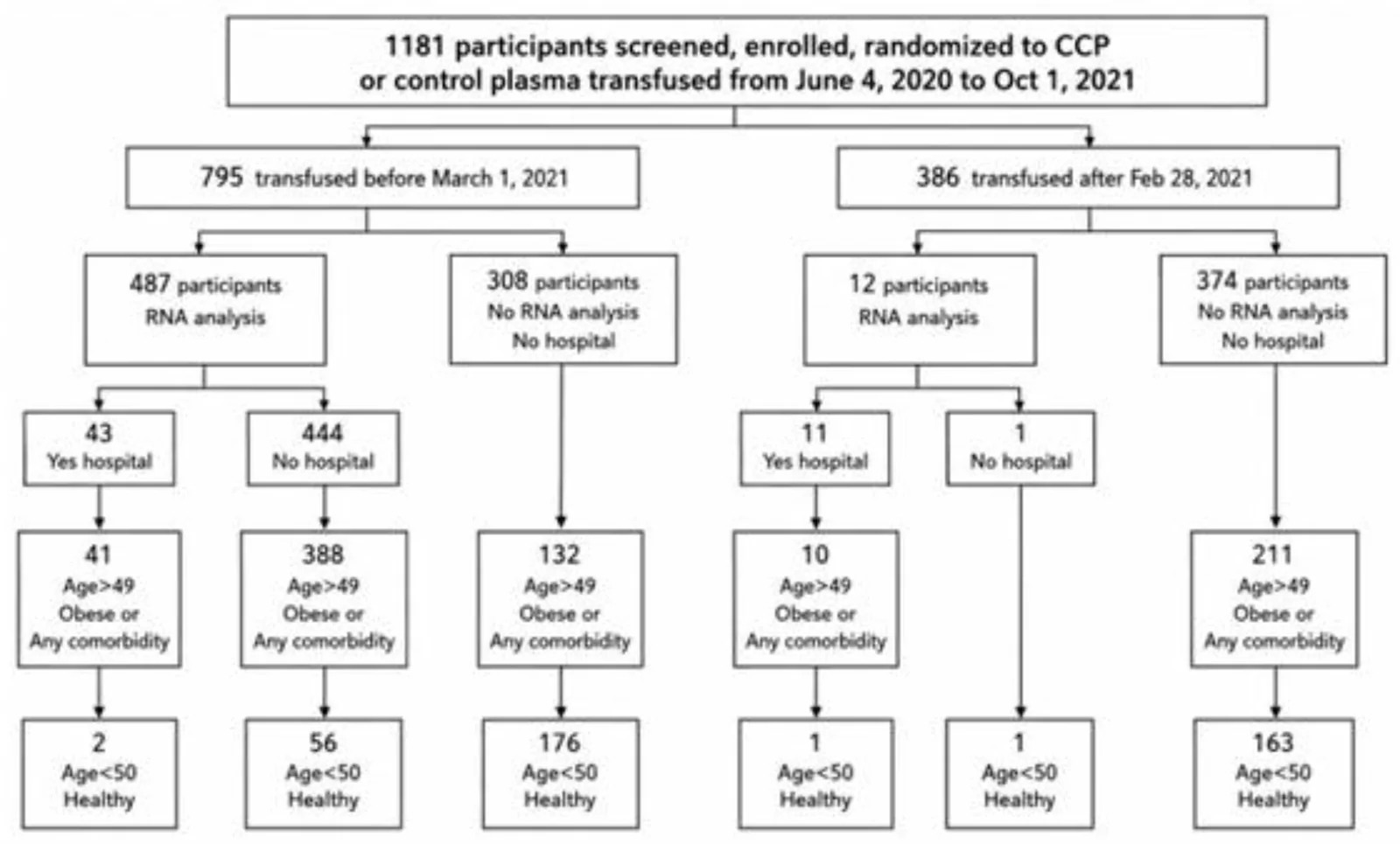
Study population segregated by transfusion period, inclusion in plasma RNA analysis, hospitalization, risk factors for severe COVID-19 progression or age below 50 and healthy.

**Figure 2.**
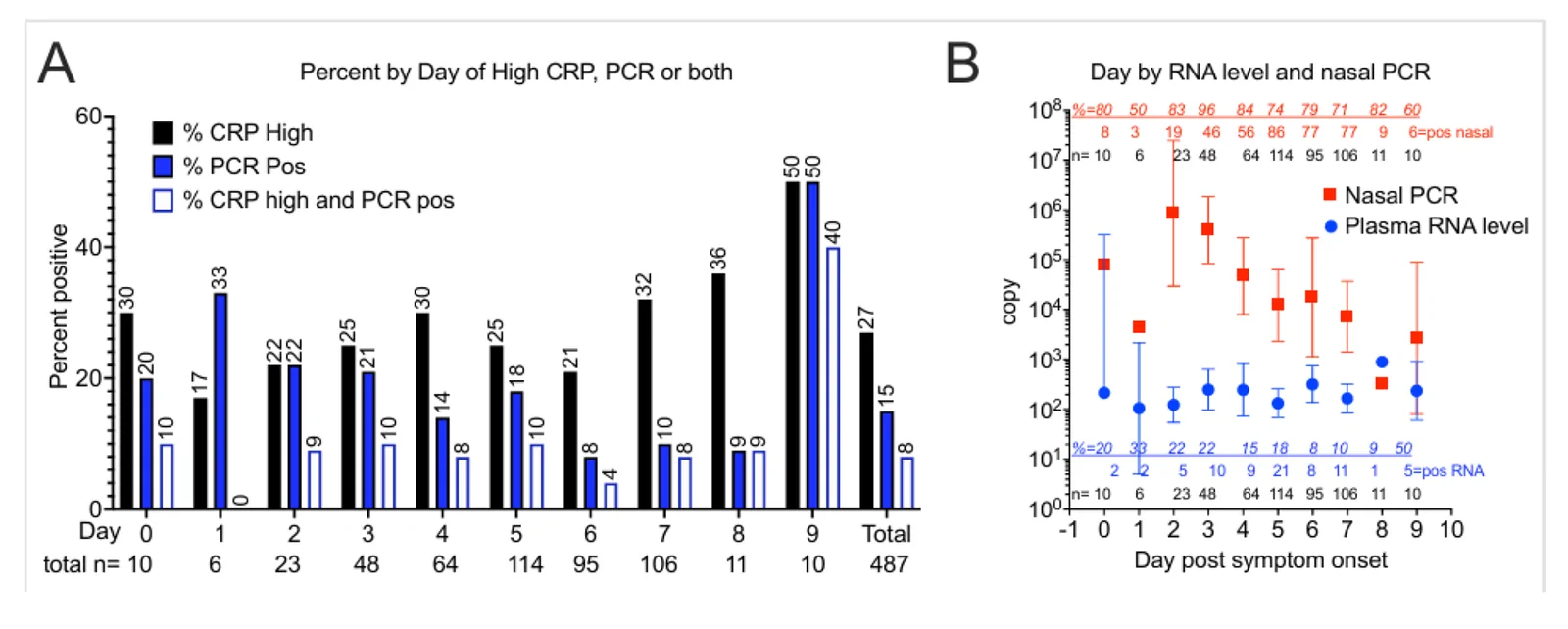
A) Time interval of symptom onset to transfusion in days and rate of high CRP, positive PCR or both; B) Plasma RNA levels (copies per mL) with nasal viral load (copies) by days to transfusion from symptom onset.

**Figure 3.**
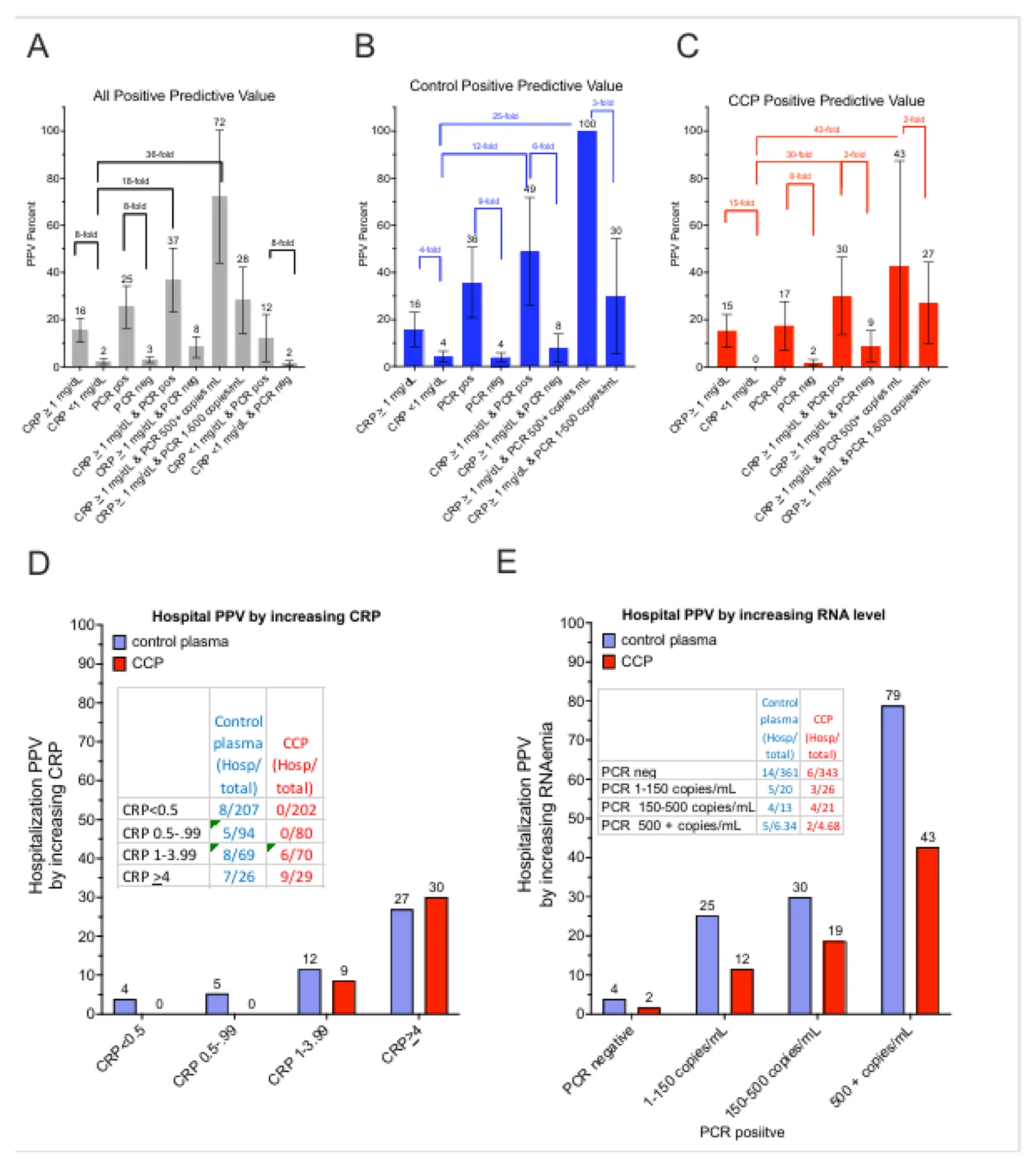
Weighted positive predictive value (PPV) for hospitalization by CRP and PCR biomarker category for A) both trial arms; B) control plasma group; C) CCP group. PPV for hospitalizations with increasing D) CRP levels and E) PCR plasma viral load by intervention arm.

**Table 1.** Comparison of the participants by transfusion period, by type of transfused plasma and by hospitalization

Variable			All		CCP		Control Plasma	

	Full study (n=1181)	Transfused before February 28, 2021 (n=795)	RNA analysis performed (n=487)	Weighted sample (n=795)	RNA analysis performed Hospital & nonhospital (n=242)	Hospital alone (n=15)	RNA analysis performed Hospital & nonhospital (n=245)	Hospital alone (n=28)
Age median (IQR)	43 (32-54)	43 (32-54)	50 (39-59)	47 (34-57)	49 (37-58)	58 (52-73)	52 (41-59)	58 (48-66)
Age ≥ 50 years	411 (35)	333 (42)	257 (53)	334 (42)	119 (49)	12 (80)	138 (56)	20 (71)
Female	675 (57)	438 (55)	262 (45)	445 (56)	120 (50)	7 (47)	142 (58)	14 (50)
Black	167 (14)	92 (12)	57 (12)	89 (11)	30 (12)	2 (13)	27 (11)	1 (4)
Hispanic	170 (14)	96 (12)	55 (11)	85 (11)	23 (10)	1 (7)	32 (13)	7 (25)
BMI ≥ 30	445 (38)	293 (37)	231 (48)	303 (38)	111 (46)	8 (53)	120 (49)	13 (46)
Age ≥ 50 years, obese or comorbidity	782 (66)	561 (71)	429 (88)	561 (71)	214 (88)	14 (93)	215 (88)	27 (96)
Any comorbidity	459 (39)	343 (43)	258 (53)	336 (42)	133 (55)	9 (60)	125 (51)	20 (71)
Hypertension	276 (23)	218 (27)	169 (35)	220 (28)	91 (38)	6 (40)	78 (32)	13 (46)
Diabetes mellitus	99 (8)	77 (10)	57 (12)	72 (9)	25 (10)	3 (20)	22 (9)	9 (32)
Symptom started 0-5 days prior to transfusion	516 (44)	361 (45)	265 (54)	445 (56)	134 (55)	4 (27)	131 (53)	21 (75)
Fully vaccinated	158 (13)	0 (0)	0 (0)	0 (0)	0 (0)	0 (0)	0 (0)	0 (0)
CRP ≥ 1mg/dL	322 (28)	198 (25)	131 (27)	193 (24)	67 (28)	15 (100)	64 (26)	15 (54)
Seronegative at screen	755 (64)	622 (78)	419 (86)	603 (76)	211 (87)	14 (93)	208 (85)	24 (86)
Hospitalization	54 (5)	43 (5)	43 (9)	43 (5)	15 (6)	15 (100)	28 (11)	28 (100)

Abbreviations: BMI, body mass index; CCP, COVID-19 convalescent plasma; CRP, C-reactive protein; IQR, interquartile range; RNA, ribonucleic acid

**Table 2.** Sensitivity and positive predictive values for CRP and plasma RNA for hospitalization

Intervention and test	Result	Hospital	No hospital	Total	Sens	Spec	PPV[95%CI]	NPV
**NON-WEIGHTED ANALYSIS**								
Both arms CRP Full Study	CRP pos	40	282	1149	74	74	12 [9-16]	98
	CRP neg	14	813					
Both arms CRP	CRP pos	30	101	487	70	77	23 [16-30]	96
	CRP neg	13	343					
Both arms RNA	RNA pos	23	51	487	53	89	31 [21-42]	95
	RNA neg	20	393					
Control Plasma CRP	CRP pos	15	49	245	54	77	23 [16-30]	93
	CRP neg	13	168					
Control plasma RNA	RNA pos	14	19	245	50	91	42 [26-59]	93
	RNA neg	14	198					
CCP CRP	CRP pos	15	52	242	100	77	22 [12-32]	100
	CRP neg	0	175					
CCP RNA	RNA pos	9	32	242	60	86	22 [9-35]	97
	RNA neg	6	195					
**WEIGHTED ANALYSIS**								
Both arms CRP	CRP pos	30	164	777	70	78	15 [10-21]	98
	CRP neg	13	570					
Both arms RNA	RNA pos	23	68	795	53	91	25 [16-35]	97
	RNA neg	20	684					
Both arms CRP pos and RNA	RNA pos	18	30.8	193.8	60	81	37 [23-52]	92
	RNA neg	12	133					
Both arms CRP pos and RNA pos	PCR500+	7	2.7	48.9	39	91	72 [37-100]	72
	PCR 1-500	11	28.2				28 [14-42]	
Control plasma CRP	CRP pos	15	80	396	54	78	16 [8-25]	96
	CRP neg	13	288				4	
Control plasma RNA	RNA pos	14	25.3	400.3	50	93	36 [20-53]	96
	RNA neg	14	347				4	
Control plasma CRP pos and RNA	RNA pos	9	9.4	95	60	88	49 [24-76]	92
	RNA neg	6	70.6				8	
Control plasma CRP pos and RNA pos	PCR500+	5	0	18.4	56	100	100 [100-100]	70
	PCR 1-500	4	9.4				30 [5-54]	
CCP CRP	CRP pos	15	84	381	100	77	15 [8-24]	100
	CRP neg	0	282				0	
CCP RNA	RNA pos	9	42.7	394.7	60	89	17 [8-29]	98
	RNA neg	6	337				2	
CCP CRP pos and RNA	RNA pos	9	21	99	60	75	30 [14-48]	91
	RNA neg	6	63				9	
CCP CRP pos and RNA pos	PCR500+	2	2.7	30.5	22	87	43 [0-100]	73
	PCR 1-500	7	18.8				27 [10-44]	

**Table 3:** Weighted comparison of the subjects whose Plasma RNA by PCR was detected and not detected on screening after weighting by inverse of selection probability

Characteristic (%)	Plasma RNA (n=91)	No Plasma RNA (n=704)	*P* value
Received CCP	52 (57)	343 (49)	.39
BMI ≥ 30	83 (91)	678 (96)	.03
Age ≥ 50 years	68 (74)	265 (38)	<.001
Male	49 (53)	302 (43)	.11
Diabetes mellitus	16 (18)	56 (8)	.01
Hypertension	49 (53)	171 (24)	<.001
Asthma	6 (7)	81 (12)	.26
Any comorbidities	64 (70)	272 (39)	<.001

CRP ≥ 1 mg/dL	48 (53)	145 (21)	<.001

Hospitalization	23 (25)	20 (3)	<.0001

Abbreviations: BMI, body mass index; CCP, COVID-19 convalescent plasma;

CRP, C-reactive protein; PCR, polymerase chain reaction; RNA, ribonucleic acid

**Table 4:** Non-weighted comparison of the participants who had COVID-19–related hospitalization between the CCP group and control plasma group

	CCP group (n=15)	Control plasma group (n=28)	*P* value*
**Parameter**			
Detectable plasma PCR, no. (%)	9 (60)	14 (50)	.75
CRP level on screening (mean)mg/dL	6.4	2.4	.002
Elevated CRP≥1 mg/dL and PCR positive, no. (%)	9 (60)	9 (32)	.11
**During Hospital Admission**			
Duration days from transfusion day to admission day, mean (IQR)	2.9 (1-4)	3.7 (2-5)	.46
Admitted within 3 days since transfusion, no. (%)	11 (73)	14 (50)	.20
Received transfusion and admitted on the same day, no. (%)	4 (27)	1 (4)	.04
Ferritin level during admission (mean)- ng/mL	1144.3	338.8	.03
CRP level during hospital admission (mean)- mg/L	11.7	9.1	.60
D-dimer level during admission (mean)- mg/L	3.7	1.1	.50
LDH level during admission (mean)- units/L	326.5	489.3	.42
Ground glass opacities either on CT scan or x-ray, no.(%)	6 (25)	15 (54)	0.59
**Hospital course/ outcomes**			
Required mechanical ventilator, bilevel positive airway			
pressure or high flow oxygen, no. (%)	2 (13)	7 (25)	.61
No supplemental oxygen needed, no. (%)	3 (20)	2 (7)	.45
ICU admission, no.(%)	4 (27)	7 (25)	.80
Remdesivir, no.(%)	8 (53)	20 (71)	.39
Dexamethasone, no.(%)	11 (73)	22 (79)	.99
COVID-19 convalescent plasma during hospitalization, no.(%)	2 (13)	9 (32)	.33
Length of stay (mean)- day	6.4	6.3	.31
Home oxygen set up on discharge, no.(%)	2 (13)	9 (36)	.23
Death, no.(%)	0 (0)	2 (11)	.54
	7 of 13		
Remained symptomatic at day 90, no.(%)	(54)	9 of 19 (47)	1.0

Abbreviation: BMI, body mass index; CRP, C-reactive protein; CT, computed tomography; ICU, intensive care unit; IQR, Interquartile range; LDH, lactate dehydrogenase; PCR, polymerase chain reaction.

* P-value estimated using Fisher’s exact test or t-test where appropriate

## Data Availability

This study is a secondary analysis of clinical data originally collected in the parent study, (Convalescent Plasma to Limit SARS-CoV-2 Associated Complications (CSSC-004) (NCT04373460) with new laboratory data on plasma. As the dataset includes demographic information from an identifiable population, individual-level data cannot be shared publicly to protect participant privacy. Summary data used in the analyses are provided within this article and within the published results of the original study.

## Highlights outpatient CRP RNAemia

- Indicates both elevated CRP and plasma RNA associated with doubling hospital risk
- Two-step biomarker approach relevant to severe disease prediction in future pandemics
- Normal CRP and plasma RNA have a high negative predictive value for hospital risk
