# Supplementary material for: Combined elevated C-reactive protein and detectable plasma RNA has predictive value for hospitalization during an outpatient COVID-19 convalescent plasma clinical trial
Source: J Clin Virol Plus. Author manuscript; Available in PMC 2026 Oct 16. (PMC13663306; doi:10.1016/j.jcvp.2026.100268)
Supplement: Supplementary file 1 [file NIHMS2212990-supplement-1.docx]

Supplementary

**COVID-10 Serologic Studies Consortium (CSSC)**

The consortium designed, implemented and analyzed the outpatient COVID-19 convalescent plasma

The members of the COVID-10 Serologic Studies Consortium (CSSC) are: Anne Arundel Medical Center (Barry R. Meisenberg); Ascada Research (Matthew Abinante, Kevin Oei); Baylor College of Medicine (Yuriko Fukuta); MedStar Georgetown University Hospital (Seble Kessaye); Johns Hopkins Center for American Indian Health (Laura L. Hammitt, Catherine G. Sutcliffe) Johns Hopkins Bloomberg School of Public Health (David J Sullivan, Bryan Lau, Atika Singh, David M. Shade, Stephan Ehrhardt, Sheriza N. Baksh, Andrew Pekosz, Sabra L. Klein, Arturo Casadevall); Johns Hopkins University (Kelly A Gebo, Shmuel Shoham, Evan M. Bloch, Christi E. Marshall, Anusha Yarava, Karen Lane, Nichol A. McBee, Amy L. Gawad, Nicky Karlen, Daniel E. Ford, Douglas A. Jabs, Lawrence J. Appel, Oliver Laeyendecker, Aaron A.R. Tobian, Daniel F. Hanley); Lifespan/Brown University Rhode Island Hospital (Adam C. Levine); Mayo Clinic, Phoenix (Janis E. Blair); MedStar Washington Hospital Center (Aarthi Shenoy); NorthShore University HealthSystem (Giselle S. Mosnaim, Thomas J. Gniadek); The Bliss Group (Michael Roth); The Next Practice Group (Colin Foster); University of California Los Angeles (Judith S. Currier); University of Alabama at Birmingham (Sonya L. Health); University of California, Irvine Health (Donald N. Forthal); University of California, San Diego (Edward R. Cachay, Elizabeth S. Allen); University of Cincinnati Medical Center (Moises A. Huaman); University of Massachusetts Worcester (Jonathan M. Gerber); University of Miami (Shweta Anjan); University of New Mexico (Jay S. Raval); University of Rochester (Martin S. Zand); University of Texas Health Science Center at Houston (Bela Patel); University of Utah Health (Emily S. Spivak, J. Robinson Singleton); Vassar Brothers Medical Center (Valerie C. Cluzet, Daniel Cruser); Wayne State University (James H. Paxton); Western Connecticut Health Network, Danbury Hospital (Joann R. Petrini, Patrick B. Broderick, William Rausch, MarieElena Cordisco); Western Connecticut Health Network, Norwalk Hospital (Jean Hammel, Benjamin Greenblatt)

Those listed in the consortium and their roles while not a contributing author to the substudy manuscript “Combined elevated C-reactive protein and detectable plasma RNA has predictive value for hospitalization during an outpatient COVID-19 convalescent plasma clinical trial” are listed with roles on the original study.

**Lawrence J. Appel**, Conceptualization, Formal Analysis and Writing – Review & Editing

**Sheriza Baksh**, Formal Analysis, Investigation, Methodology, Project Administration and Writing – Review & Editing

**Amy Gawad**, Investigation, -Methodology, Project Administration, and Writing – Review & Editing

**Jean Hammel**, Investigation, Methodology, Project Administration, and Writing – Review & Editing

**Douglas Jabs**, Conceptualization, Formal Analysis and Writing – Review & Editing

**Nicky Karlen**, Project Administration, Supervision, Validation and Writing – Review & Editing

**Christi Marshall**, Conceptualization, Investigation, Methodology, Project Administration and Writing – Review & Editing

**Joann Petrini,** Investigation, Methodology, Project Administration and Writing – Review & Editing

**Anusha Yarava**, Investigation, Methodology, Project Administration and Writing – Review & Editing

**Michael Roth,** Project Administration and Writing – Review & Editing

**Colin Foster,** Project Administration and Writing – Review & Editing

**Elizabeth Allen,** Investigation, Methodology, Project Administration and Writing – Review & Editing

**J Robinson Singleton** Investigation, Methodology, Project Administration and Writing – Review & Editing

**Full methods**

**2.1 Participant population**

This study is a follow-up secondary substudy analysis to investigate how pretransfusion CRP levels and detectable plasma SARS-CoV-2 RNA predict progression to the hospitalization primary outcome. The original outpatient, double-blind, randomized controlled trial under IND 19725 compared CCP to control plasma transfusion from June 2020 through September 2021 in 1181 confirmed COVID-19 participants enrolled within 8 days of symptom onset [1]. This subsample analyzed 487 participants including 43 of the 54 hospitalizations transfused before March 2021 with available plasma for testing.

**2.2 Study Approval**

Johns Hopkins University (JHU) served as the single-Institutional Review Board and approved the IRB protocol IRB00247590 on April 28, 2020. The protocol was also independently reviewed and approved by the Navajo Nation Health Human Research Review Board (NNR-20.376) and the Indian Health Service National IRB (N20-N-06). The IRB protocol IRB00247590 was also approved by the Department of War Human Research Protection Office. The trial was conducted in accordance with the principles of the Declaration of Helsinki, the Good Clinical Practice guidelines of the International Council for Harmonization, and all applicable regulatory requirements. Written and signed informed consent were obtained from all participants. The trial was registered on clinical trials.gov-NCT04373460. A secondary analysis protocol titled “Secondary Analysis of Outpatient COVID-19 Convalescent Plasma Studies” with IRB number IRB00415718 was also obtained.

**2.3 SARS-CoV-2 Plasma RNA analysis**

The Grifols Procleix SARS-CoV-2 transcription-mediated amplification (TMA) qualitative assay (50% LOD 2.46 copies/mL) was performed on the Panther System, using 500 μl plasma for extraction and analysis[2]. Initially 179 participants samples were analyzed by TMA, including 32 from individuals who were subsequently hospitalized. Only 179 participants were able to be evaluated by TMA due to discontinuation of reagent production by the manufacturer. Those positive for RNA by TMA (n=87; 60 nonhospitalized and 27 hospitalized) and the remaining 308 not tested in the TMA were all analyzed by qRT-PCR.

For qRT-PCR, viral RNA was extracted using QIAamp Viral RNA Mini Kit (QIAGEN). For each plasma sample, 140 µl was processed and eluted in 100 µl of water with 10 μL duplicate input into PCR or 14 μL plasma equivalents[2]. A nucleocapsid gene 73-base pair region was targeted based on an assay developed by the US Centers for Disease Control and Prevention[3]. PCR positive samples were used for plasma analysis for detectable plasma RNA as being more analogous to other published studies based upon PCR detection of SARS-CoV-2 RNA[2]. Viral RNA was amplified by one-step real-time RT-PCR using a SuperScript® III Platinum® One-Step qRT-PCR Kit (Life Technologies). For each amplification well, 10 µl (14 mL plasma equivalent) of viral RNA was added to 12.5 µl of 2X reaction mix, 0.25 µl of 100 µM forward primer nCOV_N1 For (5′-GACCCCAAAATCAGCGAAAT- 3′), 0.25 µl of 100 µM reverse primer nCOV_N1 _Rev (5′-TCTGGTTACTGCCAGTTGAATCTG-3′), 0.0375 µl of 100 µM probe nCOV_N1 -FAM (5′-ACCCCGCATTACGTTTGGTGGACC- 3′), 0.5 µl of SuperScript® III/ Platinum® Taq Mix, and 1.46 µl of nuclease-free water. Amplification conditions were: 1 cycle of 50 °C for 30 min (RT reaction) and 95 °C for 2 min (RT inactivation), followed by 45 cycles of 95 °C for 15 s and 60 °C for 1 min. RNA was run in duplicate for each sample. Positive TMA RNA was 33% (29/87) PCR positive despite using 35 times more plasma (500/14).

**2.4 Hospitalization outcomes**

Hospitalization data from discharge summaries, local principal investigator summaries and data submitted to the trial COVID-19 related hospital adjudication physician panel were reviewed for oxygen requirements, length of stay, ICU admission and laboratory studies

**2.5 Clinical data management**

Clinical data from the parent trial database were merged with laboratory measurements of SARS-CoV-2 RNA using participant study identifiers. The pretransfusion CRP was clinically measured at different study sites as a part of the original trial. Participants transfused before March 1, 2021, with available archived plasma specimens were eligible for this secondary analysis. There were no missing CRP values after selecting the 487 participants. CRP was analyzed both as a continuous variable and dichotomized using a prespecified threshold (>1 mg/dL). Plasma SARS-CoV-2 RNA was analyzed as detectable versus undetectable, with additional analyses using viral RNA >500 copies/mL. A similar study in emergency room recruited study participants randomized to CCP noted 160 positive PCR participants of 492 analyzed with median 153 and 25% and 75% 83 and 548 copies/mL[2]. Data were reviewed for completeness and consistency before statistical analysis.

**2.6 Bias mitigation.**

Selection bias was minimized by including all participants transfused before March 1, 2021, with available archived pretransfusion plasma specimens. Because plasma RNA testing was performed on a weighted subsample, inverse-probability weighting was applied to generate estimates representative of the full eligible trial population. Conduct bias was minimized because CRP and plasma SARS-CoV-2 RNA were measured from pretransfusion specimens using standardized laboratory methods, and plasma SARS-CoV-2 RNA was unavailable to the treating clinicians. Outcome assessment bias was minimized through standardized ascertainment of hospitalization outcomes using discharge summaries, research records, and review by the trial COVID-19 hospitalization adjudication panel, independent of biomarker measurements.

**2.7 Statistical analysis**

Individuals who progressed to hospitalization were selected with a probability of 1, while those who were high risk participants and those who were otherwise healthy were selected with a probability of <1. Therefore, the risk of hospitalization cannot be estimated without bias unless the sample is reweighted back to represent the original 795 participants who were at risk given the eligibility criterion of transfusion prior to March 1, 2021. For all analyses that depend on risk (e.g., positive predictive value, negative predictive value, hospital risk, etc.), the analytical sample was weighted by the inverse of the probability of selection into this subsample for plasma RNA testing[4]. This accounts for differential sampling probabilities, so that results will reflect the full eligible cohort.

The statistical relationship between hospitalization status and CRP or RNA levels was estimated by a logistic model. Because of weighted analysis, we used the robust sandwich estimator for obtaining 95% confidence intervals (CI). Furthermore, there were situations in which there was complete separation of the logistic model due to no events in one level of predictor. Therefore, we utilized Firth’s penalized logistic regression (weighted by inverse sampling probability) with 2000 iterations of bootstrap approach [5]. Analyses were performed using R and GraphPad Prism 8 (GraphPad Software).

Supplementary Figure 1 Correlation of nasal viral copy number to plasma RNA copies in A) 482 matched participant pairs including negative plasma RNAemia and B) only matched pairs with positive RNAemia.

References

[1] Sullivan DJ, Gebo KA, Shoham S, Bloch EM, Lau B, Shenoy AG, et al. Early Outpatient Treatment for Covid-19 with Convalescent Plasma. N Engl J Med. 2022;386:1700-11.

[2] Di Germanio C, Marques de Menezes EG, Clevenger R, Rines I, Durkalski-Mauldin VL, Leifer E, et al. Viral and Immune Factors Associated With COVID-19 Outcome in the C3PO Trial of Convalescent Plasma. J Infect Dis. 2025;231:1198-209.

[3] Lu X, Wang L, Sakthivel SK, Whitaker B, Murray J, Kamili S, et al. US CDC Real-Time Reverse Transcription PCR Panel for Detection of Severe Acute Respiratory Syndrome Coronavirus 2. Emerg Infect Dis. 2020;26:1654-65.

[4] Hernán MA, Hernández-Díaz S, Robins JM. A structural approach to selection bias. Epidemiology. 2004;15:615-25.

[5] Heinze G, Schemper M. A solution to the problem of separation in logistic regression. Statistics in Medicine. 2002;21:2409-19.
